# Nanohydroxyapatite-Coated Titanium Surface Increases Vascular Endothelial Cells Distinct Signaling Responding to High Glucose Concentration

**DOI:** 10.3390/jfb14040188

**Published:** 2023-03-28

**Authors:** Anderson M. Gomes, Danielle F. da Silva, Fábio J. Bezerra, Willian F. Zambuzzi

**Affiliations:** São Paulo State University (UNESP), Biosciences Institute, Campus Botucatu, Botucatu 18618-970, SP, Brazil

**Keywords:** dental implants, functional surfaces, nano-hydroxyapatite coating, diabetes, bone, angiogenesis

## Abstract

Aim: The success of dental implants depends on osseointegration can be compromised by well-known related adverse biological processes, such as infection and diabetes. Previously, nanohydroxyapatite-coated titanium surfaces (nHA_DAE) have been shown to contain properties that promote osteogenesis by enhancing osteoblast differentiation. In addition, it was hypothesized to drive angiogenesis in high-glucose microenvironments, mimicking diabetes mellitus (DM). On the other hand, the null hypothesis would be confirmed if no effect was observed in endothelial cells (ECs). Materials and methods: Titanium discs presenting the differential surfaces were previously incubated in an FBS-free cell culture medium for up to 24 h, which was, thereafter, supplemented with 30.5 mM of glucose to expose human umbilical vein endothelial cells (HUVECs, ECs) for 72 h. They were then harvested, and the sample was processed to provide molecular activity of specific genes related to EC survival and activity by using qPCR, and the conditioned medium by ECs was used to evaluate the activity of matrix metalloproteinases (MMPs). Results: Our data guaranteed better performance of this nanotechnology-involved titanium surface to this end once the adhesion and survival characteristics were ameliorated by promoting a higher involvement of β1-Integrin (~1.5-fold changes), Focal Adhesion Kinases (FAK; ~1.5-fold changes) and SRC (~2-fold changes) genes. This signaling pathway culminated with the cofilin involvement (~1.5-fold changes), which guaranteed cytoskeleton rearrangement. Furthermore, nHA_DAE triggered signaling that was able to drive the proliferation of endothelial cells once the cyclin-dependent kinase gene was higher in response to it, while the P15 gene was significantly down-regulated with an impact on the statement of angiogenesis. Conclusions: Altogether, our data show that a nanohydroxyapatite-coated titanium surface ameliorates the EC performance in a high-glucose model in vitro, suggesting its potential application in DM patients.

## 1. Introduction

The development of biomaterials to attend to the requirements of dental and medical issues has gained an important value, mainly in considering people presenting with metabolic disorders and comorbidities, such as obesity and diabetes, which are known to elevate an implant’s failure [1,2,3,4]. In this scenario, the modification of titanium surfaces, looking for biomimetic properties, appears to be an important alternative to consider in trying to overcome the insufficiency of diabetes mellitus (DM) patients in promoting angiogenesis.

Demographic data show that both men and women are living longer, concomitant with the increased number of people diagnosed with diabetes mellitus (DM) [5]. These demographic aspects bring even more attention to developing more bioactive and osteoinductive properties of osteointegrable implants. In addition, these aspects can lead to considering personalized care and offering a better quality of life, thus influencing their daily routine by restoring functions.

Recent studies show some alternatives to improve biomaterials, and a widespread proposal is for titanium suffering a dual acid-etching (DAE) [6]. However, little attention has been paid to alternative biomaterials to support bone regeneration in relation to patients suffering from a DM disorder.

DAE surfaces gather a specific surface roughness and have been shown to interact better with bone cells than machined surfaces [7]. In this way, recently, a mimic nanohydroxyapatite-coated surface has been proposed, presenting better performance in driving osteogenesis by affecting osteoblast biology (considering adhesion, proliferative and differentiate phenotypes) and, consequently, ameliorating osseointegration by enhancing the deposition of a new appositional bone [8,9]. In addition to considering osteoblasts [10,11,12,13], endothelial cells (ECs) have also been considered once they compose the microenvironment surrounding the implants [14]. ECs can positively impact the performance of dental implants by considering the molecular crosstalk between osteoblasts and endothelial cells (ECs) [15]. Importantly, we have previously shown that nanohydroxyapatite-coated surfaces promote angiogenic stimulus by requiring a PI3K/AKT signaling pathway [16]. However, very few studies have shown that this surface ameliorates bone regeneration in an osseointegration mechanism related to the behaviors of a host bone and endothelial cells.

Considering metabolic disorders, such as diabetes mellitus (DM) and obesity, there is a great challenge in developing a reliable model in vitro that is able to mimic most of the properties to improve knowledge about of these disorders. In this sense, there are several validated models available, both in vivo and in vitro, with the objective of developing the framework of DM [17]. Preclinical validated biological models in vitro need to be better investigated considering their relevance to biomaterial responses, including the application of Russell–Burch’s principles of the 3Rs (Reduction, Replacement and Refinement), prioritizing studies that use non-animal models. Nowadays, in vitro assays have shown that it is possible to mimic hyperglycemia in cell cultures. High glucose concentrations promote an increased rate of stem cell proliferation as well as cytoskeletal remodeling due to the upregulation induced by aquaporin-1 (AQP1) and have also been shown to be able to promote angiogenesis through the activation of the binding protein of tonicity enhancer (TonEBP) and possibly increasing the expression of AQP1 and COX-2 [18,19].

Thus, given the importance of ECs in the peri-implant tissue healing process and the low angiogenic potential in DM, the objective of this study was to better evaluate the involvement of these cells by responding to a medium containing a high concentration of glucose enriched with titanium and subjected to both DAE or nHA_DAE. Since intracellular signaling pathways are able to indicate the quality of cell interaction, we focused our study on investigating the molecular mechanism that guarantees EC activities in response to biotechnologically modified surfaces. On the other hand, a null hypothesis would be confirmed if no effect was observed in endothelial cells (ECs).

In summary, the novelty of this study is in the capacity of nanohydroxyapatite-coated surfaces to promote better microenvironments for ECs adaptation in high-glucose models in vitro mimicking diabetes mellitus (DM) by regulating the crucial intracellular signaling networks responsible for the maintenance of cell survival, cytoskeletal rearrangement and ECM remodeling. 

## 2. Material and Methods

### 2.1. Titanium Alloys, Reagents and Titanium-Enriched Medium Preparation

The titanium discs were generously donated by the SIN (São Paulo, SP, Brazil). The Gotaq qPCR master mix (A6002) was purchased from PROMEGA (Madison, WI, USA, EUA). In order to prepare the titanium-enriched medium, both the dual acid etching (DAE) treating surfaces (named w_DAE) and nanohydroxyapatite-coated titanium surfaces (named nHA_DAE) with the experimental alloys (*n* = 6) were incubated in cell culture media (RPMI) without FBS for up to 24 h at 37 °C, 5% CO_2_, and 95% humidity 0.2 g/mL (*w*/*v*), with a slight modification of ISO 10993-5:2016. The titanium-enriched medium was expected to contain molecules released from titanium-modified surfaces and might have affected the biology of EC, as this study considered microenvironments mimicking diabetes. 

### 2.2. Experimental Design

**Figure 1:**
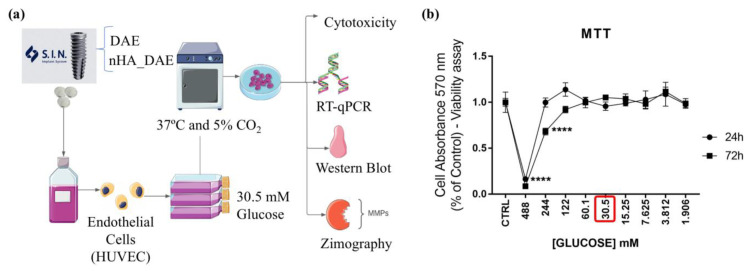
Outline and workflow of this study. Previously, the titanium discs were incubated in FBS-free cell culture medium for up to 24 h. Thereafter, the conditioned media were collected and sufficient glucose was added to reach a concentration of 30.5 mM (red frame), in addition to supplementation with 10% FBS. Then this medium was later used to expose ECs for up to 72 h, and thereafter, the conditioned medium was harvested for zymography assay, and the cells were properly prepared to allow the analysis of gene activities using qPCR technology (**a**). Cell viability assay was performed to identify better concentration of glucose to be used in this study. The concentration of glucose tested here ranged from 488 mM of glucose to 1.906 mM, wherein the cell viability was measured by the MTT approach (**b**). **** *p* < 0.001. Each experiment was carried out in triplicate (*n* = 3).

Titanium discs were subjected to both of the surfaces evaluated in this study, dual acid-etched (DAE) and nanohydroxyapatite-coated surfaces (nHA_DAE) [20,21]. Technically, to obtain the titanium-enriched medium from the samples, 0.6 cm diameter discs were maintained in conic tubes containing cell culture medium 0.2 g/mL *w*/*v*, without fetal bovine serum (FBS) for up to 24 h as recommended by ISO 10993-5:2016 (Figure 1a). The conditioned medium was later used to expose ECs for up to 72 h, wherein the cells were harvested to allow gene expression. Figure 1b shows a graph depicting the cytotoxic effect of glucose concentration ranging from 488 mM to 1.906 mM in ECs. Concentrations of glucose above 10 mM are analogous to the diabetic condition within the cell culture system. A concentration of 30.5 mM was used to expose the ECs for up to 72 h (Figure 1a).

### 2.3. Cell Line and Culture Conditions

Human Umbilical Vein Endothelial Cells (HUVEC) were purchased from American Type Culture Collection (ATCC, Manassas, VA, USA) and properly maintained in an RPMI medium containing antibiotics (100 U/mL penicillin, 100 mg/mL streptomycin) and 10% (*v*/*v*) Fetal Bovine Serum (Nutricell, Campinas, SP, Brazil), which was then incubated at 37 °C, 5% CO_2_, and 95% humidity.

### 2.4. Cell Viability Assay

Despite the literature already stating that a concentration of 30.5 mM of glucose is enough to characterize high-glucose Medium, we decided to test cell viability responding to a range of glucose concentrations: 1.906 mM to 488 mM of glucose. Initially, the cells were cultured in 75 cm^3^ flasks until they reached semi-confluence. After this period, the HUVECs were then trypsinized and seeded (1 × 10^5^ cells/well) into 96-well microplates. One day after seeding, the cells were treated with a medium containing a high concentration of glucose (30.5 mM). Cell viability was checked considering the time of exposition of 24 h or 72 h. Thereafter, the MTT solution was prepared from the salt of the compound (≥97.5%, Sigma-Aldrich, San Louis, MO, USA) in an FBS-free cell culture medium at a final concentration of 1 mg/mL. The exposition medium was then replaced by a fresh medium prepared with the MTT salt for up to 3 h. After this period, the medium was removed, and 100 µL of absolute ethanol was placed in each well. Cell viability was later estimated by measuring the absorbance in a microplate reader (SYNERGYHTX multi-mode reader, Biotek, Winooski, VT, USA) at a 570 nm wavelength.

### 2.5. Experimental DM Condition—High-Glusose In Vitro

An experimental model that uses high glucose to mimic a DM condition was proposed in this study. In this sense, many protocols have been discussed about the adequate experimental workflow to mimic a diabetic condition, mainly considering a high glucose concentration. Of course, considering the limitations of an in vitro model, we simulated this condition by exposing the endothelial cells to a concentration of 30.5 mM D-glucose. Considering the effects of the osmolarity background of the high glucose concentration on endothelial cell signaling, we intended to expose a control culture group to high mannitol concentrations. Importantly, this testing medium was properly supplemented with 10% FBS and antibiotics.

### 2.6. mRNA Isolation and RT-qPCR Analysis

After exposing the endothelial cells to high glucose concentrations and a titanium-enriched medium for up to 72 h, we focused on properly preparing the samples to allow the transcript (mRNA) analysis. Thus, properly exposed endothelial cells during the 72 h were harvested, and the total RNA was isolated using Ambion TRIzol Reagent (Life Sciences-Fisher Scientific Inc., Walthan, MA, USA) and thereafter treated with DNase I (Invitrogen, Carls-band, CA, USA). cDNA synthesis was performed with a High-Capacity cDNA reverse transcription kit (Applied Biosystems, Foster City, CA, USA) according to the manufacturer’s instructions. qPCR was carried out with a final total of 10 μL of reaction volume containing PowerUpTM SYBRTM Green Master Mix 2× (5 μL) (Applied Biosystems, Foster City, CA, USA), 0.4 μmol L^−1^ of each primer, 200 ng of cDNA and nuclease-free H_2_O. Data were expressed as relative amounts of the transcripts normalized with three housekeeping genes (ß-ACTIN, GADPH and 18S genes), using the cycle threshold (ct) method. Primers and experimental details are described in Table 1.

### 2.7. Gelatin Proteolysis-Based Zymography

During the experimental model, aliquots of the conditioned medium from endothelial cells responding to high glucose and titanium (DAE or nHA_DAE) were collected to estimate the extracellular matrix (ECM) remodeling stimulus, by measuring the MMP activities through the gelatin-base proteolysis zymography technology. After obtaining the samples, they were pooled by centrifugation at 14,000 rpm for up to 15 min to avoid cell debris, and the protein concentration was determined by the Lowry method [22]. The same amount of protein was resolved into a 12% polyacrylamide gel containing 4% gelatin. The gelatinolytic activity was evaluated in the resolved proteins. Thereafter, their renaturation using Triton X-100 aqueous solution (2% *w*/*v*)was incubated for 18 h in a proteolysis buffer (Tris-CaCl_2_) at 37 °C and then stained with a Coomassie Blue R-250 dye solution 0.05% for 3 h, when the gels were washed in a 30% methanol (*v*/*v*) and 10% glacial acetic acid solution (*v*/*v*). The opposite staining was obtained in the gels at the exact point of the gelatinolytic activity (bands) of metalloproteinases 2 (MMP2~62 kDa) and 9 (MMP9~84 kDa), and then they were analyzed using the software ImageJ (Bethesda, MD, USA), following a previous study [23].

### 2.8. Statistical Analyses

Results were represented as mean ± standard deviation (SD). The samples assumed a normal distribution with *p* < 0.05, considered statistically significant, and *p* < 0.001, considered highly significant. In the experiment where there were >2 groups, we used a two-way ANOVA with multiple comparisons in order to compare all pairs of groups. In this case, the significance level was considered when alpha = 0.05 (95% confidence interval). The software used was GraphPad Prism 7 (GraphPad Software, San Diego, CA, USA).

## 3. Results

In order to evaluate the performance of endothelial cells (ECs) responding to nHA_DAE in microenvironments mimicking DM, ECs were harvested to allow the analysis of specific genes related to cell adhesion and survival, as well as their capacity in remodeling the extracellular matrix (ECM).

**Figure 2:**
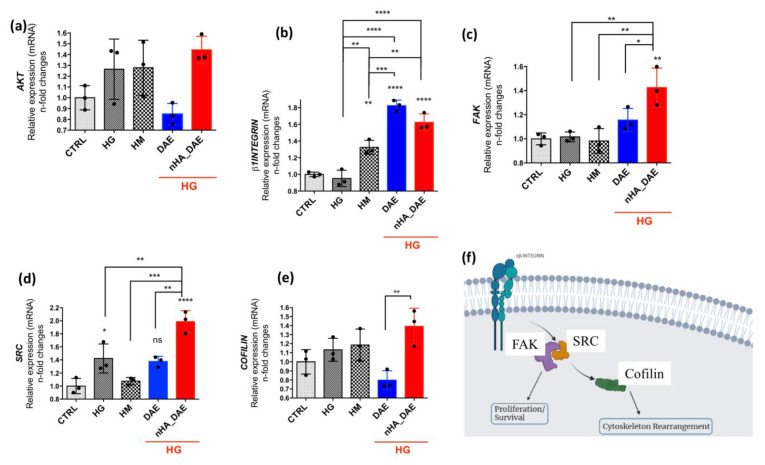
Cytoskeleton rearrangement is stimulated by nHA_DAE in a high-glucose microenvironment. (**a**) INTEGRIN-β1, (**b**) FAK, (**c**) SRC, (**d**) AKT (**e**) COFILIN, (**f**) Schematization of signaling pathways proposed in this study. High-glucose (HG) and high-mannitol (HM) cultures were considered control groups. Both DAE and nHA_DAE were maintained in a presence of HG. Differences were considered significant when * *p* < 0.05, ** *p* < 0.01, *** *p* < 0.001 **** *p* < 0.0001, and when compared with the CTRL or among groups. The experiment was carried out in triplicate (*n* = 3).

Regarding the genes related to cell adhesion and cytoskeleton rearrangement, our data show that there is a significantly higher expression of ß1-INTEGRIN in response to DAE and nHA_DAE, although a high mannitol concentration has also promoted a slightly higher expression of this gene (Figure 2a). Furthermore, the FAK gene was higher expressed in EC responding to nHA_DAE in microenvironments mimicking DM (Figure 2b), and a very similar profile was found considering SRC (Figure 2c). This signaling upon integrin activation seems to require COFILIN, which was also higher in response to nHa_DAE (Figure 2e). Importantly, the AKT gene remains unchanged in response to titanium surfaces, as well as considering the endothelial cells responding to high glucose and high mannitol (Figure 2d). Figure 2f shows a diagram reinforcing the involvement of FAK/SRC/COFILIN upon integrin activation, and it is expected to maintain survival signaling in endothelial cells.

**Figure 3:**
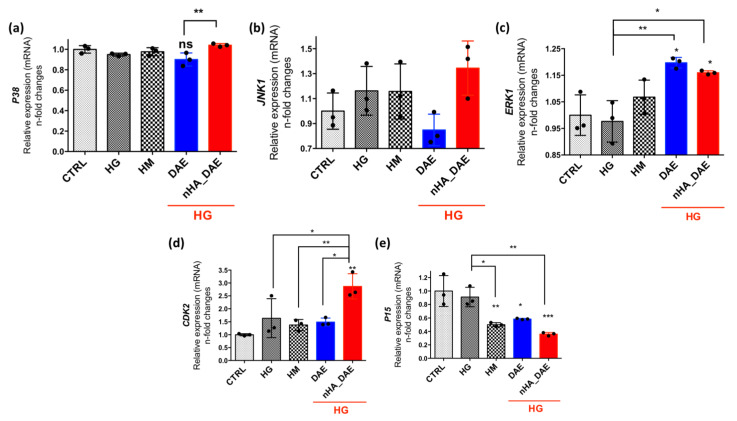
nHA_DAE requires the activation of genes related with survival and proliferative phenotypes. MAPK-P38 (**a**), MAPK-JNK1 (**b**), and MAPK-ERK1 (**c**) genes were evaluated in this study. Considering the proliferative phenotype of endothelial cells responding to titanium-enriched medium, CDK2 (**d**) and P15 (**e**) genes were evaluated. For qPCR analysis, we considered GAPDH, β-ACTIN and 18S as housekeeping genes. Differences were considered significant when * *p* < 0.05, ** *p* < 0.01, *** *p* < 0.001 when compared with the CTRL or between the groups. The experiment was carried out in triplicate (*n* = 3).

Additionally, the MAPKs were investigated, and it is known that these proteins are related to cell survival. Considering the MAPKs P38 (Figure 3a), ERKs (Figure 3b) and JNKs (Figure 3c), nHA_DAE promotes higher modulations, with significance considering P38 and JNK, while ERK was slightly higher but without static significance. Importantly, the capacity of the endothelial cells to respond to nHA_DAE was further investigated by considering their ability to drive the activation of genes related to proliferative phenotypes, such as CDK2 and P15. Our data show that endothelial cells responding to nHA_DAE require a significantly higher expression of the CDK2 gene (~3-fold changes, Figure 3d), while the P15 gene was decreased in the groups exposed to high glucose or high mannitol (Figure 3e).

**Figure 4:**
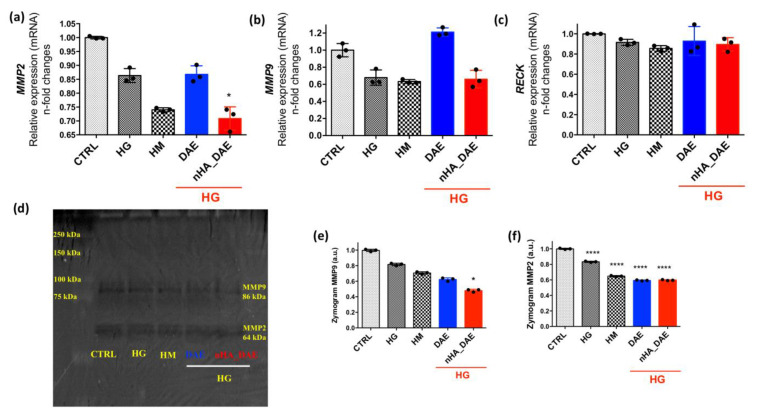
ECM remodeling was investigated by gelatin proteolysis-based zymography. In order to evaluate ECM remodeling, we firstly evaluated gene expression of MMP2 (**a**), MMP9 (**b**) and RECK (**c**) genes. Thereafter, the activities of MMPs were also evaluated by zymography assay (**d**–**f**). GAPDH, β-ACTIN and 18S genes were used as housekeeping genes. Statistical differences were considered when compared to the control group or between the groups: * *p* < 0.05 and **** *p* < 0.0001. The experiment was carried out in triplicate (*n* = 3).

Finally, ECM remodeling was investigated by considering the transcriptional profile of gelatinases A and B, MMP2 and MMP9, respectively. Our data show there is a lower expression of MMP2 in response to nHA_DAE (Figure 4a). The behavior of the MMP9 gene expression was slightly lower but without significance (Figure 4b). Additionally, the RECK gene was also investigated here; however, there were no differences when considering the tested groups (Figure 4c). The behavior of the gene expression was likely to have been reflected in the MMP2 and nine activities, as shown in Figure 4d–f.

## 4. Discussion

Dental implant survival is dependent on successful osseointegration and can be compromised by well-known related biological processes such as infection or metabolic upset that may adversely affect the osseointegration [1]. Diabetes may be heightening the critical dependence of the biology of osseointegration by compromising the bone metabolism driven by bone cells [2]. In turn, diabetes is conceived as a chronic disease related to high levels of circulating glucose as a result of the body being unable to effectively use the insulin that it produces. Over the last few decades, it has been intensively discussed that diabetic patients are susceptible to an increased chance of developing periodontitis and tooth loss [24], culminating in the high risk of dental implant failure, also because they are associated with delayed wound healing [25], the prevalence of microvascular diseases [26] and impaired response to infection [27]. Thus, it is clear that there is relevance in finding a bioactive surface able to enhance angiogenesis in the peri-implant surrounding tissue formed during the wound healing process. In order to evaluate this topic, we have investigated the capacity of nanohydroxyapatite-coated titanium surfaces (here called nHA_DAE) in promoting the enhancement of endothelial cell activity, mainly considering survival and proliferative profiles in an in vitro high-glucose mimicking diabetes model. Our data guarantee better performance of this nanotechnology-involved titanium surface to this end.

Firstly, we have noticed a higher expression of genes related to endothelial cell adhesion, where the ß1-INTEGRIN, FAK and SRC genes were higher expressed in response to nHA_DAE. These three proteins interact with each other in molecular platforms structured by Paxilin [28], guaranteeing the better performance of cell–substratum adhesion and avoiding signaling pathways related to anoikis drought and the loss of substrate adhesion of anchorage-dependent cells [29]. The involvement of these complex molecules during cell adhesion also promotes the activation of signaling pathways related to cell survival via PI3 kinase/Akt signaling [30]. In this way, we have clearly shown that nHA_DAE promotes the activation of a PI3K/AKT in endothelial cells [16], and now it appears to be involved in response to a high glucose concentration mimicking diabetes model. With special attention to β1integrin, it is related to composing heterodimer with α1, which binds to collagen [31].

Complimentarily, the SRC kinase has been listed upstream to cytoskeleton rearrangement of eukaryotes [32,33,34,35], and it gains even more relevance in endothelial cells responding to shear stress by driving the process of actin polymerization in a very dynamic manner and can be associated with endothelial cell permeability; this control is relevant to considering the endothelial cells as a semipermeable layer lining all blood vessels [36], regulating blood vessel formation and contributing to barrier function. Herein, our data support the hypothesis of a cytoskeleton rearrangement in response to nHA_DAE by relating COFILIN to the downstream of this pathway upon integrin activation once COFILIN is widely discussed in modulating the actin polymerization [33,37]. The composition of endothelial cell–matrix adhesion complexes is associated with these signaling pathways, and the remodeling of ECM can be associated with the disruption of this linkage.

Considering the endothelial cell-–matrix adhesion, ECM remodeling gains relevance to the integrity of the endothelium. Here, we have investigated the ability of endothelial cells to respond to nHA_DAE in high-glucose microenvironments mimicking DM in modulating matrix metaloproteinases (MMPs) activities. Our data show that there is a reduction in ECM remodeling in this condition by endothelial cells responding to nHA_DAE.

Another important topic investigated here was the proliferative phenotype of endothelial cells responding to nHA_DAE. Our data show that the CDK2 gene expression was higher in response to nHA_DAE in the DM mimicking model, and it supports the nHA_DAE in being related to cell cycle progression in endothelial cells, hypothesizing the enhancing of angiogenesis in the peri-implant microenvironment, and it can overcome poor vascular networks in diabetic patients. CDK2 gene expression has been understood as being markedly reduced in endothelial cells in confluency and can be inhibited by the activity of p27 [38]. In addition, a number of these CDK2 physiological inhibitors have been reported, including p21, p27, p16, P15, p19, and p57 [39]. Our data show a repressive behavior of the P15 gene in endothelial cells responding to nHA_DAE that ensures the prevalence of proliferative phenotype in responding to these nanotechnological-related surfaces.

## 5. Conclusions

Altogether, our data show that a nanohydroxyapatite-coated titanium surface ameliorates EC performance in a high-glucose model in vitro, and it supports its application in DM patients. This is expected to enhance peri-implant wound healing by considering its ability to promote angiogenesis and an adequate vascular network surrounding the dental implants that is a prerequisite to bone regeneration. 

## Figures and Tables

**Table 1:** Expression primers sequences and PCR-related cycle conditions.

Gene	Primer	5′-3′ Sequence	Reaction’s Conditions
INTEGRIN-β1	Forward	GCCGCGCGGAAAAGATGAA	95 °C—15 s; 60 °C—30 s; 72 °C—30 s
	Reverse	TGCTGTTCCTTTGCTACGGT	
SRC	Forward	CAACACAGAGGGAGACTGGT	95 °C—15 s; 60 °C—30 s; 72 °C—30 s
	Reverse	AGCTTCTTCATGACCTGGGC	
COFILIN	Forward	TGTGCGGCTCCTACTAAACG	95 °C—15 s; 60 °C—30 s; 72 °C—30 s
	Reverse	TCCTTGACCTCCTCGTAGCA	
ERK	Forward	ACCAGGCTCTGGCCCACCCAT	95 °C—15 s; 60 °C—30 s; 72 °C—30 s
	Reverse	GCAGCGCCTCCCTTGCTAGA	
P38	Forward	GAGAACTGCGGTTACTTA	95 °C—15 s; 60 °C—30 s; 72 °C—30 s
	Reverse	ATGGGTCACCAGATACACAT	
JNK	Forward	AAAGGTGGTGTTTTGTTCCCAGGT	95 °C—15 s; 60 °C—30 s; 72 °C—30 s
	Reverse	TGATGATGGATGCTGAGAGCCATT	
AKT	Forward	CCAGCCTGGGTCAAAGAAGT	95 °C—15 s; 60 °C—30 s; 72 °C—30 s
	Reverse	TCTCCTCCTCCTCCTGCTTC	
CDK2	Forward	CTTTGCTGAGATGGTGACTCG	95 °C—15 s; 60 °C—30 s; 72 °C—30 s
	Reverse	GCCTCCCAGATTCCTCATGC	
P15	Forward	GGGACTAGTGGAGAAGGTGC	95 °C—15 s; 60 °C—30 s; 72 °C—30 s
	Reverse	CATCATCATGACCTGGATCGC	
MMP2	Forward	AGCTCCCGGAAAAGATTGATG	95 °C—15 s; 60 °C—30 s; 72 °C—30 s
	Reverse	CAGGGTGCTGGCTGAGTAGAT	
MMP9	Forward	CACGCACGACGTCTTCCA	95 °C—15 s; 60 °C—30 s; 72 °C—30 s
	Reverse	AAGCGGTCCTGGCAGAAAT	
FAK	Forward	TCAGCTCAGCACAATCCTGG	95 °C—15 s; 60 °C—30 s; 72 °C—30 s
	Reverse	CTGAAGCTTGACACCCTCGT	
RECK	Forward	TGCAAGCAGGCATCTTCAAA	95 °C—15 s; 60 °C—30 s; 72 °C—30 s
	Reverse	ACCGAGCCCATTTCATTTCTG	
PI3K	Forward	ACTCTCAGCAGGCAAAGACC	95 °C—15 s; 60 °C—30 s; 72 °C—30 s
	Reverse	ATTCAGTTCAATTGCAGAAGGAG	
ß-ACTIN	Forward	ACAGAGCCTCGCCTTTGC	95 °C—15 s; 60 °C—30 s; 72 °C—30 s
	Reverse	GCGGCGATATCATCATCC	
GAPDH	Forward	AGGCCGGTGCTGAGTATGTC	95 °C—15 s; 60 °C—30 s; 72 °C—30 s
	Reverse	TGCCTGCTTC ACCACCTTCT	
18S	Forward	CGGACAGGATTGACAGATTGATAGC	95 °C—15 s; 60 °C—30 s; 72 °C—30 s
	Reverse	TGCCAGAGTCTCGTTCGTTATCG	

## Data Availability

The data that support the findings of this study are available from the corresponding author upon reasonable request.
